# Intensity-modulated Radiotherapy Versus Volumetric Modulated Arc Therapy in Head and Neck Cancers: A Comparative Analysis of Compliance, Toxicities and Dosimetric Parameters

**DOI:** 10.7759/cureus.86143

**Published:** 2025-06-16

**Authors:** Sanyamita Jain, Piyush Kumar, Pavan Kumar, Jitendra Nigam, Silambarasan N.S, Rashika Sachan

**Affiliations:** 1 Radiation Oncology, Shri Ram Murti Smarak Institute of Medical Sciences, Bareilly, IND; 2 Medical Physics, Shri Ram Murti Smarak Institute of Medical Sciences, Bareilly, IND

**Keywords:** compliance, dosimetry, head and neck cancers, imrt, toxicity, vmat

## Abstract

Introduction

Intensity-modulated radiotherapy (IMRT) is now a standard technique to treat head and neck cancers. Another advanced technique - volumetric-modulated arc therapy (VMAT) - is gaining popularity due to rapid treatment delivery, which allows patient comfort and reduced intrafraction motion. The present study aims to compare the compliance, dosimetric parameters and acute toxicities between both techniques in head and neck cancer patients.

Materials and methods

One hundred de-novo patients of head and neck cancers presenting to our department and planned to be treated by definite chemoradiation either due to medical inoperability, patient preference or anatomical inaccessibility for surgery were selected. Patients were assigned to one of the treatment groups quasi-randomly in a 1:1 ratio, with odd-number participants planned to be treated by IMRT (Group I) and the even-number patients planned to be treated by VMAT (Group II). Contrast-enhanced computed tomography (CECT) scan of the head and neck region was used for simulation and radiotherapy planning, and target volumes were delineated along with organs at risk (OARs). The dose prescription was at 70 Gy in 35 fractions at 2 Gy/fraction for five days a week, delivered with concurrent chemotherapy (injection Cisplatin @35 mg/m^2^ weekly). Concurrent chemotherapy was avoided in septuagenarian patients, those with Cisplatin ineligibility or in early glottic cancer patients.

Planning target volume (PTV) dosimetric parameters calculated were: V66.5Gy (%), D2% (Gy), D50% (Gy), D98% (Gy), Dmax (Gy), Dmean (Gy), homogeneity index, conformity index and monitor units (MUs). Dose constraints to OARs given were Dmax (Gy) to planning organ at risk volume (PRV) brainstem, PRV spine, optic chiasma, optic nerve, lens and mandible; Dmean (Gy) to both parotids, cochlea, PseudoOAR and lips. Though brachial plexus and dysphagia aspiration-related structures (DARS) were prospectively contoured, they were not included in the dose planning.

Patients were assessed weekly during the treatment for oral mucositis, radiation-induced dermatitis and haematological toxicity and were followed up at least for six months from the day of the completion of treatment. Statistical analysis was performed using IBM SPSS version 22, and p-value <0.05 was considered significant.

Results

Amongst PTV parameters for IMRT vs VMAT, D2 (71.9±0.58 vs 73.2±0.66 p=0.02), Dmax (74.09±0.6 vs 74.5±1.11; p=0.04) and MUs (2002.4 vs 604.78, p=0.0002) showed statistical significance, contributing to decreased beam on time during treatment delivery, which was better in the VMAT arm. OAR dosimetry was lower in VMAT for right cochlea (20.6 vs 15.97, p=0.07), right lens (2.69 vs 2.01, p=0.04), left optic nerve (6.05 vs 3.21, p=0.04) and lips (19.05 vs 14.88, p=0.004) but statistically significant. Other dosimetric parameters and dosimetry of OARs did not show any statistical difference. The RTOG grade 3-4 skin reactions between IMRT and VMAT were found to be comparable (p=0.82), but not statistically significant. The RTOG grade 3-4 oral mucositis between IMRT and VMAT were also found to be comparable (p=0.63), but not statistically significant.

Conclusion

VMAT is better than IMRT in terms of lesser MUs delivered but with comparable PTV, OAR dosimetry and acceptable acute toxicities. The long-term follow-up in terms of secondary malignancies needs to be evaluated.

## Introduction

The treatment for squamous cell cancers of the head and neck region is site- and stage-specific. In early stages, the treatment is generally through a single modality, either surgery or radiotherapy. The treatment for locally advanced head and neck cancers involves either surgery followed by adjuvant radiation with or without concurrent chemotherapy as guided by pathological features or definitive chemoradiation. Radiotherapy in the head and neck cancers can be delivered by conventional or conformal techniques. Conventional radiotherapy for head and neck cancers has significant acute and late morbidity and conformal techniques like three-dimensional conformal radiotherapy (3D-CRT) and intensity-modulated radiotherapy (IMRT) improve dose delivery while preserving the neighbouring normal tissues and organs at risk (OARs). IMRT exploits inverse planning and optimization algorithms to provide treatment fields with different beam intensities, tailored to fit the anatomy of each patient, creating precise three-dimensional dosage distribution. Volumetric modulated arc therapy (VMAT) is now routinely available where a continuous arc is employed. In VMAT, the multi-leaf collimators (MLCs), variable dose rate and gantry speed allow the radiation to be adjusted as the gantry of the linear accelerator revolves around the patient.

Radiotherapy techniques for head and neck cancer have been ever evolving with the aim of decreasing the side effects of head-neck radiation namely mucositis, dysphagia and odynophagia, which may need treatment to be discontinued or extended, which may even lead to untoward outcomes in terms of disease control [1,2]. Long-term side effects like Xerostomia, or the subjective experience of a dry mouth caused by hypofunction of the salivary glands may result in oral pain, prolonged dry mouth and trouble speaking and swallowing, hampering the quality of day-to-day life.

IMRT has been in clinical use since the 1990s and due to its therapeutic advantage is now the standard of care. Compared to static-beam IMRT, rotational VMAT is supposed to decrease treatment delivery times with at least similar plan quality [3]. The greatest benefit of VMAT is rapid delivery, allowing improved patient comfort, reduced intrafraction motion and increased patient load on machine. Therefore, IMRT and VMAT have emerged as key techniques that offer enhanced dose conformity and sparing of healthy tissues compared to conventional radiotherapy. Despite increasing global use of VMAT, particularly in resource-rich centers, IMRT remains the standard in many settings but comes with planning complexity. However, there are concerns about low-dose spill in VMAT as well. Comparative clinical and dosimetric data remain important to guide decision-making in varied clinical environments. The merits of VMAT need to be weighed against the demerits to justify its utilization. The objective of this study is to compare IMRT and VMAT in head and neck cancers focusing on patient compliance in terms of treatment adherence, acute toxicities and dosimetric parameters.

## Materials and methods

Study setting and description

This randomized prospective interventional cohort study was conducted at the Department of Radiation Oncology, Shri Ram Murti Institute of Medical Sciences, Bareilly, Uttar Pradesh, India, between August 2022 and January 2024. The study included treatment-naive patients of head and neck malignancies, who were treated by definitive chemoradiation after a multi-disciplinary tumour board discussion depending on the subsite and inoperability of the primary malignancy.

Inclusion and exclusion criteria

All patients >18 years, with histologically proven squamous cell carcinoma of head and neck region, stages I-IVA (American Joint Committee on Cancer (AJCC), 8th edition), who were planned for definitive chemoradiotherapy due to medical inoperability, patient preference or anatomical inaccessibility for surgery, Karnofsky performance status >70, normal haemogram, renal function tests, liver function tests and two-dimensional echocardiography were included in the study. Post-operative and metastatic cases, patients with prior or synchronous malignancy and previously treated patients with radiotherapy were excluded from the study population. 

Sample size and randomization

The sample size was calculated using a complete enumeration method where all the patients meeting the inclusion criteria and presenting in the study period were included in the study. Quasi-randomized allocation, based on patient number parity, was used and the patients were then randomly assigned in a 1:1 ratio into two groups using a simple randomization method where odd-number participants were planned to be treated by IMRT (Group I) while even-number patients were planned to be treated by VMAT (Group II).

Radiotherapy planning and technique

The patients were immobilized on a base plate in a neutral or extended neck position as indicated, using a fixed five-point thermoplastic cast, with individualized supportive neck rest. Contrast-enhanced CT (CECT) and simulation scan radiotherapy planning (RTP) of 1.5 mm slice thickness were obtained in a supine position with three radio-opaque fiducial markers aligned in a single CT slice. These images were then transferred through Digital Imaging and Communications in Medicine (DICOM-CT) into the Eclipse treatment planning system (TPS) (Version 13.6, Varian Medical System, Inc., Palo Alto, CA, USA).

Delineation of Structures

Delineation of gross disease was described as gross tumour volume (GTV), microscopic spread of primary gross tumour as clinical target volume primary (CTVp) and draining nodal region related to primary clinical target volume nodal (CTVn). We followed guidelines for the delineation of neck node level for head and neck tumours as described by Biau et al. [4]. CTV Final included both CTV primary and CTV nodal. A symmetrical margin of 5 mm was taken from CTV to account for patient setup error as per the institutional protocol and defined as planning target volume (PTV). The delineation of organs at risk structures (OARs) included left and right parotid glands, spinal cord, brain stem, eyes, lens, optic chiasma, optic nerve, cochlea (right and left), lips, dysphagia aspiration-related structures (DARS), brachial plexus and mandible. They were delineated as per Danish Head and Neck Cancer Group (DAHANCA) [5] guidelines. An isotropic expansion of 5 mm was given for planning risk volume (PRV) spine and 3 mm planning organ at risk volume (PRV) for brainstem as per institution protocol. Pseudo-OAR was defined as the unspecified region between the PTV and OAR where the dose is often dumped during planning.

Dose Prescription and Organ at Risk Evaluation

All the patients were planned either for IMRT or VMAT technique as per their assignment to the respective groups. The total prescription dose was 70 Gy in 35 fractions, five days a week. The DAHANCA recommendations on fractionation (i.e., no six-fraction-per-week acceleration or treatment duration ≤48 days) were not followed due to logistic constraints and in consideration of patient tolerance as per the institutional protocol.

The ideal planning objective was to achieve a minimum dose of 95% of the whole PTV and a maximum dose of 107% relative to the prescribed dose. The following OAR dosimetric constraints were prescribed at the time of treatment planning: PRV brainstem: Dmax <54 Gy; PRV spinal cord: Dmax <50 Gy; mandible: Dmax <70 Gy; optic nerve: Dmax <54 Gy; parotid gland: Dmean <26 Gy; cochlea: Dmean <45 Gy as per the Quantitative Analyses of Normal Tissue Effects in the Clinic (QUANTEC) recommendations [6] to minimize the risk of radiation-induced acute and late side effects of the organs at risk. PseudoOAR was given a dose prescription of Dmean <30 Gy. DARS and brachial plexus were prospectively contoured but not given dose constraints at the time of treatment prescription.

IMRT Planning Technique

Coplanar seven to nine fields, using 6 MV energy, at the dose rate of 400 cGy/minute were used, around isocentre using isotropic gantry angles and adjusted slightly to avoid the beam entry through OARs during the optimization grid size of 2.5 mm and photon optimizer algorithm were used for IMRT planning. Normal tissue objective was used for controlling the dose to undefined normal tissue. The dose coverage minimum and maximum required for PTV and dose tolerance to OARs were introduced as upper, lower and mean objectives of the respective organs. Monitor unit (MU) calculation was done using anisotropic analytical algorithm after the next step of fluence optimization. Then the plan was evaluated by two methods: isodose coverage and dose-volume histogram (DVH). The plan was then compared with an alternate plan to improve treatment quality.

VMAT Planning Technique

The VMAT plans were performed using two to four coplanar arcs (clockwise rotation from 181˚ to 179˚ and anti-clockwise rotation from 179˚ to 181˚ using 6 MV energy at the dose rate of 600 cGy/min. During planning, pseudo-contours are created and included in the optimization to control cold and hot spots to improve plan quality. The plan was optimized using photon optimizer algorithm. Normal tissue objective was used for controlling the dose to undefined normal tissue. The dose coverage minimum and maximum required for PTV and dose tolerance to OARs were introduced as upper, lower and mean objectives of the respective organ. Monitor unit calculation was done using an anisotropic analytical algorithm after the next step of fluence optimization. The completed plan was evaluated by isodose coverage and DVH. 

Dosimetric Assessment

The doses that the PTV and OAR received were determined from cumulative dose-volume histograms, as recommended in the International Commission on Radiation Units and Measurements (ICRU) Report 83 [7]. The PTV dosimetric parameters evaluated to assess plan quality included V66.5, Dmax, Dmean, D2%, D50%, D98%, homogeneity index (HI) [8] and Paddick’s conformity index (pCI) [9].

Homogeneity index: HI was calculated using the following formula [8]:

$HI = \frac{{D_{2\%} - D_{98\%}}}{{D_{50\%}}}$

where D2%, D50%, and D98% are the absolute dose delivered to 2%, 50%, and 98% of the PTV, respectively. An HI value of zero indicates a homogenous distribution. 

Conformity index: Conformity index (CI) is a measure of the degree of conformity of the absorbed dose distribution to the PTV. In this work, Paddick’s CI [9] was used for evaluation:

$(TVPIV)^2 / (TV * PIV)$

where TVPIV, TV and PIV are the prescribed isodose volume over the target volume, the target volume and the prescription isodose volume, respectively.

Chemotherapy protocol 

As per the institutional protocol, concurrent chemotherapy was delivered @ 35mg/m^2^ weekly in patients with normal haemogram, renal function tests, liver function tests, normal PTA, normal 2D echocardiogram (echo), creatine clearance >60ml/min and age <70 years who were planned for definite chemoradiation due to medical inoperability, patient preference or anatomical inaccessibility for surgery. Chemotherapy was also avoided in patients with early glottic cancer. A three-weekly regimen of Cisplatin @ 100mg/m^2^ was not followed due to poor tolerability in our patient population.

Clinical response assessment

The patients were assessed for objective tumour response according to WHO criteria [10]: (1) complete response (CR): total tumour regression for at least four weeks; (2) partial response (PR): 50% or more reduction in the product of two major perpendiculars of the measurable tumour for at least four weeks; (3) stable disease (SD): less than 50% or more reduction to less than 25% increase in cross-product; and (4) progressive disease (PD): growth of measurable tumour by 25% or more or appearance of a new lesion.

Assessment of toxicity

Patients were assessed weekly during chemoradiation for acute radiation reactions. Radiation toxicity was assessed by RTOG (Radiation Therapy Oncology Group) acute morbidity scoring criteria [11] including oral mucositis and radiation-induced dermatitis. Haematological toxicity was also assessed on a weekly basis.

Statistical analysis and follow-up

The statistical software used was IBM SPSS version 22 (IBM Corp, Armonk, NY). The continuous variables were presented in terms of mean±standard deviation. Normality test was done. Independent t-test was used after verifying the assumption of normality. A p-value <0.05 was considered significant at a 5% level of significance for a one-tailed t-test. The patients were followed up at least for a period of six months from the day of the completion of treatment.

## Results

A total of 100 patients were recruited for the study, with 50 patients in each arm. The patient characteristics of the study population are presented in Table 1. The patient characteristics showed the mean age in Group I was 53.64 years and that in Group II was 57.68 years. The male-to-female ratio in Group I was 22:3 and that in Group II was 23:2 The most common site in the study was oropharynx (38%) followed by buccal mucosa (12%) and glottic larynx (12%).

**Table 1:** Patient characteristics of the study population A summary of patient characteristics between both arms in the study population. GBS: Gingiva-buccal sulcus; RMT: retromolar trigone.

Patient Characteristics	IMRT, n=50 (n%)	VMAT, n=50 (n%)
Sex		
Male	44 (88%)	46 (92%)
Female	6 (12%)	4 (8%)
Addiction		
Tobacco chewing + alcohol	17 (34%)	17 (34%)
Tobacco smoker	16 (32%)	19 (38%)
Tobacco chewer	12 (24%)	12 (24%)
Alcohol consumption	13 (26%)	13 (26%)
Smoking + alcohol	22(44%)	26(52%)
Site		
Buccal Mucosa	9 (18%)	3 (6%)
Tongue	5 (10%)	6 (12%)
Glottic Larynx	4 (8%)	8 (16%)
Alveolus	1 (2%)	0 (0%)
GBS	1 (2%)	0 (0%)
RMT	2 (4%)	1 (2%)
Oropharynx	15 (30%)	23 (46%)
Hard Palate	1 (2%)	0 (0%)
Supraglottic Larynx	9 (18%)	2 (4%)
Hypopharynx	2 (4%)	7 (14%)
Maxilla	1 (2%)	0 (0%)
T stage		
T1	1(2%)	1 (2%)
T2	15 (30%)	9 (18%)
T3	16 (32%)	20 (40%)
T4	18 (36%)	20 (40%)
N stage		
N0	6 (12%)	5 (10%)
N1	4 (8%)	10 (20%)
N2	14 (28%)	24 (48%)
N3	26 (52%)	11 (22%)
Group Stage		
I	1 (2%)	1 (2%)
II	5 (10%)	5 (10%)
III	4 (8%)	6 (12%)
IVA	22 (44%)	26 (52%)
IVB	18 (36%)	12 (24%)

Dosimetric assessment

PTV and OAR dosimetric parameters from this study are presented in Tables 2 and 3, respectively. The PTV parameters (Table 2) such as V95 (%), D2 (Gy), D98 (Gy), D50 (Gy), conformity index and homogeneity index were compared between the two arms. In our study, IMRT had a lower V959(%) than VMAT (98.66±0.01 vs 98.9±0.02; p=0.08) while VMAT had a higher Dmax (74.09±0.6 vs & 74.5±1.11; p=0.04), which was statistically significant. D2 was higher in the VMAT arm (71.9±0.58 vs 73.2±0.66), which was highly statistically significant (p=0.02) owing to the low-dose spill region known to be present with continuous gantry rotation in VMAT. The D98 was comparable in both arms (67.14 vs 67.01; p=0.69). Both plans produced comparable plans in terms of homogeneity index (0.06±0.02 vs 0.08±0.01; p=0.25) and conformity index (0.74±0.11 vs 0.75±0.95; p=0.75). The average MUs (Tables 2 and 4) in the IMRT arm were 2002.4±814.7 and those in the VMAT arm were 604.78±202.9 (p=0.0002), which also showed statistical significance, contributing to decreased beam on time during treatment delivery.

**Table 2:** PTV Dosimetry Summary: VMAT and IMRT A summary of dosimetric parameters between both arms. P value was calculated using unpaired t-test, which was found to be statistically significant, in Dmax (0.04), D2 (0.002) and MUs (0.0002). IMRT: intensity-modulated radiotherapy; VMAT: volumetric modulated arc therapy; HI: homogeneity index; CI: conformity index; MU: monitor unit.

Technique	IMRT (Mean and SD)	VMAT (Mean and SD)	P value
1. V95	98.66±0.01	98.9±0.02	0.08
2. Dmax (Gy)	74.09±1.06	74.5±1.11	0.04
3. Dmean (Gy)	70.2±0.54	70.2±0.64	0.81
4. D2 (GY)	71.9±0.58	73.2±066	0.002
5. D50 (GY)	70.3±0.57	70.4±0.58	0.15
6. D98 (GY)	67.14±1.88	67.01±1.44	0.69
7. HI	0.06±0.02	0.08±0.1	0.25
8. CI	0.74±0.11	0.75±0.95	0.75
9. MUs	2002.4±0.11	604.25±207.48	0.0002

**Table 3:** Dosimetry Summary OAR: VMAT and IMRT arms. A summary of OARs between both arms, P value was calculated using unpaired t-test, which showed that while both modalities could achieve all dose constraints, VMAT achieved lower constraints which were statistically significant in left optic nerve (p =0.04), right lens (p=0.04), left cochlea (p=0.05) and lips (p=0.004). IMRT: intensity-modulated radiotherapy; VMAT: volumetric modulated arc therapy; PRV: planning risk volume; DARS: dysphagia aspiration-related structures.

OARS	IMRT (Mean and SD)	VMAT (Mean and SD)		P value
1. PRV Brainstem	41.32±10.08		40.13±12.01	0.08
2. PRV SpinalCord	46.73±5.03		46.6±2.6	0.86
3. Optic chiasma	5.88±8.8		3.7±3.8	0.12
4. Optic nerve (R)	5.30±8.05		3.5±4.4	0.17
5. Optic nerve (L)	6.057±9.68		3.21±2.56	0.04
6. Lens (L)	2.640±2.22		2.06±1.46	0.1
7. Lens (R)	2.69±2.1		2.01±1.26	0.04
8. Mandible	71.62±4.41		70.3±10.67	0.41
9. Parotid (L)	39.8±13.0		38.2±10.26	0.43
10. Parotid (R)	41.88±14.3		38.59±10.8	0.2
11. Cochlea (L)	28.13±14.0		20.66±12.6	0.05
12. Cochlea (R)	20.66±13.57		15.97±11.9	0.07
13. Lips	19.05±13.5		14.88±11.7	0.004
14. DARS	62.90±6.7		64.78±5.95	0.14
				15. Brachial plexus	71.48±3.5		69.91±5.47	0.09
16. PseudoOAR	24.83±11.8		23.58±12.53						0.6

When critical OAR dosimetric parameters were evaluated (Table 3) between the two arms, a comparable dosimetry between most OARs was observed. Dmax of PRV brainstem (Gy) was 41.32±10.08 (IMRT) vs 40.13±12.01 (VMAT) (p=0.08). Dmax of PRV spinal cord (Gy) was 46.73±5.03 vs 46.6±2.6 (p=0.86), Dmax of optic chiasma (Gy) was 5.88±8.8 vs 3.7±3.8 (p=0.12). Dmax of right optic nerve (Gy) was 5.30±8.05 vs 3.5±4.4 (p=0.17). Dmax of the left lens (Gy) was 2.64±2.22 vs 2.06±1.46 (p=0.10). Dmax of mandible (Gy) was 71.62±4.41 vs 70.3±10.67 (p=0.41). Dmean of the left parotid (Gy) was 39.8±13.0 vs 38.2±10.26 (p=0.43). Dmean of the right parotid (Gy) was 41.88±14.3 vs 38.59±10.8 (p=0.20). Dmean of the right cochlea (Gy) 20.66±13.57 vs 15.97±11.9 (p=0.07), all of which did not show any statistical significance. While in Dmax of the left optic nerve (Gy) was 6.057±9.68 vs 3.21±2.56 (p=0.04), Dmean of the left cochlea was 28.13±14.0 vs 20.66±12.6 (p= 0.05). VMAT also had lower doses in Dmax of the right lens (Gy): 2.69±2.1 vs 2.01±1.26 (p=0.04), Dmax of optic chiasma (Gy) was 5.88±8.8 vs 3.7±3.8 (p=0.12). The Dmean of the lips (Gy) was significantly lower in the VMAT arm 19.05±13.5 vs 14.88±11.7 (p=0.004), which could also be because of a higher percentage of patients of buccal mucosa cancer in the IMRT arm and the anatomic proximity of the PTV with lips.

**Table 4:** MUs in IMRT and VMAT arm The higher number of MUs in the IMRT arm. IMRT: intensity-modulated radiotherapy; VMAT: volumetric modulated arc therapy; MU: monitor unit.

Sno.	IMRT	VMAT
1	1336	832
2	1446	467
3	1521	490
4	1547	363
5	1419	490
6	1413	498
7	1615	915
8	1317	483
9	1609	438
10	1805	459
11	1210	482
12	1437	401
13	1436	493
14	1642	464
15	1634	480
16	780	439
17	1286	484
18	1611	424
19	1112	809
20	1071	420
21	3281	480
22	1413	517
23	1570	536
24	1605	393
25	2657	396
26	2801	833
27	4203	481
28	2877	863
29	2184	476
30	1300	869
31	2741	878
32	3201	482
33	1666	905
34	1249	903
35	1596	490
36	2963	441
37	2109	970
38	2766	934
39	2645	971
40	3157	423
41	2828	462
42	3650	890
43	2342	574
44	998	538
45	3328	927
46	2976	421
47	1679	753
48	2074	717
49	2881	485
50	1135	900

Clinical response, compliance and toxicity assessment

Toxicity Assessment

The RTOG grade 3-4 skin reactions between IMRT and VMAT were found to have a comparable p- value (=0.82), calculated using the chi-square method, which was not statistically significant. The RTOG grade 3-4 oral mucositis between IMRT and VMAT were also found to be comparable (p= 0.63), calculated using chi-square method, but was not statistically significant. The weekly haematological toxicity was comparable between both arms.

Compliance

Compliance was measured in terms of adherence to treatment protocol, that is, completion of chemoradiation in 49 days. Any deviation was considered to be non-compliance. Concurrent chemotherapy compliance in IMRT and VMAT arm was 58% vs 68%, while 24% patients defaulted radiotherapy in the IMRT arm vs 14% patients in the VMAT arm. The mortality in IMRT arm was 16% and that in VMAT was 6%.

Response Assessment

The patients were assessed for objective tumour response according to WHO criteria (Table 5), which was found to be comparable, with no statistically significant difference in the treatment response between IMRT and VMAT.

**Table 5:** WHO response to treatment A summary of WHO response criteria to treatment in both arms, which were comparable. IMRT: intensity-modulated radiotherapy; VMAT: volumetric modulated arc therapy.

WHO Response Criteria	IMRT, n=50 (n%)	VMAT, n=50 (n%)	P value
Complete Response	28 (56%)	30 (60%)	0.2
Partial Response	6 (12%)	6 (12%)	0.3
Progressive Disease	1 (2%)	1 (2%)	0.3
Not Classified	7 (14%)	10 (20%)	0.2

## Discussion

IMRT and VMAT are advanced precision radiotherapy techniques and have established superiority over conventional techniques in attaining conformal dose distribution and OAR sparing. But the debate of IMRT being better than VMAT or vice versa is still ongoing. Literature shows heterogenous results. While some studies show the superiority of IMRT over VMAT, others have found the two comparable and some found VMAT to produce better plan indices.

PTV parameters

Vanetti et al. [12] evaluated 29 patients with carcinoma of the oropharynx, hypopharynx and larynx and reported a higher value of the maximum dose for Rapid Arc or VMAT plans when compared to IMRT. The target coverage and homogeneity were improved with Rapid Arc-2 when compared with Rapid Arc-1 and IMRT, while the conformity index was comparable. Similarly, in the study led by Wiehle et al. [13], of a total of 15 patients, 13 patients were treated by IMRT and two patients were treated by VMAT for carcinoma of the oropharynx, hypopharynx, larynx, nasopharynx and oral cavity. Cervical lymphadenopathy unknown primary (CLUP) was planned to a dose of 70 Gy. The VMAT plans produced a higher dose in the PTV. In our study, IMRT had a lower V95% than VMAT (98.66±0.01 vs 98.9±0.02, p=0.08), which showed a trend towards statistical significance, but with a higher Dmax in VMAT (74.09±0.6 vs 74.5±1.11, p=0.04), which was statistically significant and higher D2 in the VMAT arm (71.9±0.58 vs 73.2±0.66), which was highly statistically significant (p=0.02). This can be attributed to dose dumping in VMAT due to continuous gantry rotation, making the benefit debatable since both IMRT and VMAT achieved clinically acceptable plans, which were delivered, but low-dose region increases the risk of secondary malignancies.

In the systemic review by Buciuman and Marcu [14], which included 31 studies of head and neck cancers and prescription of 70 Gy delivered by simultaneous integrated boost (SIB) or sequential technique, the dosimetric advantages of VMAT over IMRT were evaluated. For this, only 13 studies could be analysed, though the prescribed dose differed from case to case. It varied between 66, 70 and 77 Gy for high‐risk PTV, 66, 60 and 59.4 Gy for intermediate-risk PTV and 56 and 54 Gy for low‐risk PTV, variations that do not allow for a significant statistical analysis. A majority of the studies all succeeded in showing the superiority of VMAT in PTV coverage, while Clemente et al. [15] and Stieler et al. [16] where both patient populations were treated by sequential techniques achieved lower values for conformity index for VMAT plans. Two studies that enrolled 20 head and neck cancer patients, each with doses (54‐70 Gy) in 33 fractions, were treated by SIB. While Lee et al. [17] found no significant difference in conformity index between VMAT and IMRT, Lu et al. [18] observed that both conformity index and heterogeneity index were higher for IMRT. Ali [19] also evaluated 11 patients retrospectively with advanced head and neck tumours, which were planned using SIB. Both IMRT and VMAT were planned to a prescription of 33 fractions with five fractions weekly. The prescribed doses were 70 Gy, 59.4 Gy and 54 Gy for the high, intermediate, and low risk. A comparable HI in IMRT and VMAT was observed, but VMAT showed superiority in terms of conformity index (0.76 vs 0.68) and monitor units (589 vs 1989). This was contrary to our study where SIB was not used. Instead, the entire PTV was prescribed at a dose of 70 Gy in 35 fractions, at 2 Gy per fraction. Comparable PTV parameters between IMRT and VMAT were found in terms of heterogeneity index and conformity index. Studies [20-22] have clinically found higher acute toxicity, grade 3 dysphagia and higher treatment breaks (7% vs. 0%) when SIB was combined with concurrent chemotherapy when compared to sequential boost. We therefore did not opt for SIB in our study.

In the study by Singh et al. [23], 21 cases of head-and-neck cancers were planned by sliding window IMRT, step-and-shoot IMRT and double Arc VMAT for dosimetric comparison. They concluded that target volume coverage was comparable with IMRT and VMAT (p>0.05) with no significant differences in homogeneity index and conformation index. This was similar to our study where PTV dosimetric parameters such as conformity Index and homogeneity index were comparable in both arms (homogeneity index: 0.06±0.02 vs 0.08±0.01; p=0.25 and conformity index: 0.74 ±0.11 vs 0.75 ±0.95; p=0.75). 

Another study comparing IMRT and VMAT in head and neck cancers two years before the current study, but in a post-operative setting, with a prescription of 60Gy in 30 fractions without concurrent chemotherapy conducted by Aparajeeta et al. [24] concluded that both IMRT and VMAT plans showed comparable dosimetry, but IMRT plans exhibited slightly better conformity index (1.14±0.09 vs 1.29±0.13, p=0.002), which was contrary to our study where the conformity index was 0.74±0.1 vs 0.75±0.95 (p=0.75). This could be attributed to more experience acquired in producing better quality VMAT plans by the medical physicist over time, demonstrating that plan quality can even vary due to experience.

Monitor units 

IMRT and VMAT both produce acceptable plans, but it is established in the literature [13-20] that the number of MUs in IMRT are significantly higher than in VMAT. Since the amount of scattered radiation that reaches the healthy tissues is directly proportional to the number of MUs delivered, a lower number of MUs equates to a dose reduction to the distant healthy tissues, which is lower in VMAT because of continuous gantry rotation. We had similar results where the average MUs in IMRT arm were 2002.4±0.11 and that in VMAT arm were 604.78±202.9 (p=0.0002), which also showed statistical significance.

Organs at risk

Parotid 

IMRT was popularized over three-dimensional conformal radiation therapy (3DCRT) due to its ability to achieve parotid sparing, which decreases the risk of xerostomia, which can also be extrapolated in VMAT. Vanetti et al. [12] reported a decreased mean parotid dose in VMAT. Similarly in the systemic review by Buciuman and Marcu [14], except one study, all the other studies reported a lower mean dose for parotid glands in the case of VMAT plans. In our study, while neither modality achieved the mean dose constraint, VMAT had slightly lower parotid doses, but it was not statistically significant (p=0.4). This is because in most contours, there was a PTV and parotid contour overlap and PTV coverage was given priority over parotid in order to avoid a geographical miss.

Cochlea

Dosimetric parameters for the cochlea were reported in a few studies. Studentski et al. [25] reported very similar cochlea doses for the two techniques: 27 Gy for VMAT vs 28 Gy for IMRT whereas in our study we found that VMAT had superior dosimetry than IMRT in the left optic nerve (Gy): 6.057±9.68 vs 3.21±2.56 (p=0.04), left cochlea (Gy): 28.13±14.0 vs 20.66±12.6 but not in the right cochlea (Gy): 20.66±13.57 vs 15.97±11.9 (p=0.07) and right optic nerve (Gy): 5.30±8.05 vs 3.5±4.4 (p=0.17). Both IMRT and VMAT plans achieved dose constraints, which were also clinically implemented, or patients received concurrent chemotherapy with cisplatin, which has a known side effect of ototoxicity. The doses need to be as low as possible because higher doses may precipitate hearing loss in these patients receiving concurrent chemotherapy. Moreover, higher doses also pose a challenge when planning for reirradiation in head and neck cancers. 

Spinal Cord

While some studies [26] demonstrated no statistical difference between the Dmax of spinal cord, others [27] have reported a decreased dose of the spinal cord with VMAT. In our study, a comparable dosimetry between IMRT and VMAT was seen in PRV spinal cord (Gy): 46.73±5.03 vs 46.6±2.6 (p=0.86).

Mandible 

Kumar et al. [28] and Fung et al. [29] demonstrated a reduction in maximal dose to mandible in VMAT when compared to IMRT, contrary to our study where both IMRT and VMAT produced a comparable mandible dosimetry (Dmax (Gy): 71.62±4.41 vs 70.3±10.67 (p=0.41)), with slightly lower values in VMAT, which were not statistically significant. 

Lip

The dosimetry of the lip has not been discussed in most studies, but our study demonstrated that while both IMRT and VMAT achieved dose constraints, VMAT plans had lower mean dose (14.8 vs 19.5, p=0.004), which holds clinical relevance since acute mucositis increases with concurrent chemotherapy. Most patients early in the course of radiotherapy develop mucositis, especially at the angle of the mouth, which also alters alimentation. Poor nutrition further exacerbates acute reactions, which may eventually lead to treatment breaks, prolonging the overall treatment time (OTT). These treatment breaks, if they occur especially in the fifth to sixth week of radiotherapy, may promote the proliferation of resistant clones, leading to accelerated repopulation and eventually to treatment failure. 

Jaiswal et al. [26] demonstrated no statistical significance in acute radiation toxicity, though a trend toward lesser toxicity was observed in VMAT, but Holt et al. [30] found reduced oral cavity dose from 39.4 to 36.7 Gy (p<0.001) in VMAT when compared to IMRT, while Smet et al. [31] demonstrated Rapid Arc VMAT had higher skin toxicity but was better tolerated in terms of oral mucositis and dysphagia.

Gour et al. [32] demonstrated that delineating RVR and prescribing dose constraints reduced the clinical severity of oral mucositis, indicating its potential benefit in mitigating it. Khattar et al. [33] assessed whether delineating unspecified structures could reduce oral mucosa dose without compromising treatment plan quality, naming the structures “pseudo OAR” and prescribing dose constraints (Dmean≤30 Gy) to these structures. In our study, both IMRT and VMAT dose constraints to PseudoOAR could be achieved (24.83±11.8 vs 23.58±12.53, p=0.60) with comparable results, but the percentage of grade III/IV oral mucositis was higher (20% vs 14%) in IMRT than in the VMAT arm and the grade III/IV skin reactions were comparable in both IMRT and VMAT (30% vs 28%). One patient each in both arms developed grade 4 skin reactions. Ferreira et al. [34] emphasizes that radiotherapy compliance is primarily hampered by treatment-related side effects. This can also be demonstrated in our study where more patients in the IMRT arm defaulted treatment than in the VMAT arm (24% vs 14%) and did not complete treatment. This could be attributed to higher percentage of acute reactions in the IMRT arm (grade III/IV oral mucositis was 20% in IMRT and 14% in VMAT).

DARS

Amongst the few studies that compared dysphagia-associated structures and brachial plexus between the two arms, Studenski et al. [25] presented a decrease of the mean dose for larynx from 41 Gy with IMRT to 40 Gy with VMAT. Two studies held at our institute, first by Upadhyay et al. [35], suggest a significant benefit in reducing dysphagia and improving the quality of life for head-and-neck cancer patients undergoing chemoradiotherapy when dose constraints to DARS are achieved. Similarly, the second study by Agarwal et al. [36], demonstrated the feasibility of DARS sparing in IMRT, especially in laryngeal and hypopharyngeal cancer in distant constrictor muscles from PTV. Our study compared the dosimetry of DARS in IMRT and VMAT and found that IMRT had a lower dose to DARS (62.90±6.7 vs 64.78±5.9) but was not statistically significant. This could be due to higher patient cohort of oropharyngeal and laryngeal cancers in the VMAT arm as the larynx and base of the tongue are DARS structures.

Brachial Plexus

A study by Khattar et al. [37] done at our institute, which evaluated the dosimetric parameters of brachial plexus in head and neck cancers, showed that due to high dose received by the brachial plexus in head and neck cancer patients, it should be considered as an organ at risk. Alexander et al. [38] further demonstrated that the nodal stage and the primary location (namely oropharynx and hypopharynx) had a significant impact on the Dmax of brachial plexus more than the T stage of the tumour because it defines the nodal contouring. In our study, most patients (approximately 90%, in both arms) had a positive nodal disease and a large number of the patient population in the study cohort had primary oropharyngeal or hypopharyngeal cancer and it was found that while both IMRT and VMAT failed to spare the brachial plexus. VMAT resulted in a lower dose to the brachial plexus than IMRT (71.48±3.5 vs 69.91±5.47; p=0.09) with no statistical significance.

Treatment response 

In our study, 56% in IMRT and 60% in VMAT displayed a complete response to treatment, but 12% of the patients in both arms had a partial response, 2% in each arm had progressive disease and 14% in IMRT and 20% in VMAT had a response which could not be classified. This was not statistically significant.

Implications and actions needed

IMRT has comparable dosimetric parameters of PTV and critical OARs to VMAT and both techniques can achieve the prescription provided. Dose constraints should be prescribed to brachial plexus, DARS and pseudo-OAR as a means to improve quality of life. In our study, dose to these structures was notably lower in the VMAT arm, but constraints were not prescribed before treatment planning. The feasibility of DARS-sparing IMRT has been demonstrated in multiple studies [35,36]. This can also be achieved using the VMAT technique.

Strengths and limitations

The strength of our study is that dosimetric parameters of brachial plexus, DARS and “pseudoOAR” were also compared along with other OARs, which are not routinely assessed. Moreover, along with dosimetric parameters, acute toxicities and compliance were also compared between the two arms, implying the similarities between the two treatment modalities. The limitations of the study is that blinding was not done during influence toxicity scoring, which can induce bias in the assessment of toxicity. Moreover, the study has a short follow-up period and therefore long-term morbidity couldn't be compared in the two arms. VMAT due to its low dose region may pose an increased risk of secondary cancers. Although radiation-induced head and neck sarcoma (RISHNN) represents merely 1% of all head and neck sarcomas, it is challenging to diagnose and manage. Another shortcoming of our study is the lack of clinical testing for radiation-induced brachial plexopathy and DARS-associated swallowing dysfunction on follow-up visits. Moreover, constraints to these structures were not prescribed before the initiation of treatment. The study also has no stratification by tumor site/stage and patient-reported outcomes have also not reported.

## Conclusions

This study found that IMRT and VMAT provide comparable dosimetric outcomes for both the planning target volume (PTV) and surrounding critical organs at risk (OARs). Given these similarities, VMAT can be a suitable alternative in centers with high patient volumes, provided the technology is available. VMAT’s shorter treatment times and lower monitor units (MUs) make it especially advantageous for elderly or claustrophobic patients. Therefore, both techniques are effective, and the choice between IMRT and VMAT should be guided by individual patient needs and institutional capabilities.
